# Massive Hemoptysis From Concurrent Pulmonary Tuberculosis, Necrotizing Methicillin-Resistant Staphylococcus aureus Pneumonia, and Pneumocystis jirovecii Coinfection Revealing Idiopathic CD4 Lymphocytopenia

**DOI:** 10.7759/cureus.105652

**Published:** 2026-03-22

**Authors:** Ismael Palacio, Oren G Nedjar, Zaneh Kahook, Miguel Barros, Adriana A Penate Armesto, Juan C Pantoja, George Michel, Lourdes Bosch

**Affiliations:** 1 Internal Medicine, Larkin Community Hospital, Miami, USA; 2 Internal Medicine, Nova Southeastern University Dr. Kiran C. Patel College of Osteopathic Medicine, Davie, USA; 3 Neurology, Nova Southeastern University Dr. Kiran C. Patel College of Osteopathic Medicine, Davie, USA; 4 Pulmonary and Critical Care, Larkin Community Hospital, Miami, USA

**Keywords:** cellular immune dysfunction, hiv-negative immunodeficiency, idiopathic cd4 lymphocytopenia, massive hemoptysis, mrsa pneumonia, necrotizing pneumonia, opportunistic pulmonary infections, pneumocystis jirovecii pneumonia, pulmonary tuberculosis

## Abstract

Idiopathic CD4 lymphocytopenia (ICL) is a rare and underrecognized immunodeficiency characterized by persistent CD4⁺ T-cell depletion in the absence of HIV infection or other identifiable causes. Patients are often diagnosed only after presenting with severe opportunistic infections. Massive hemoptysis is a life-threatening complication typically attributed to a single pulmonary pathology, and the coexistence of multiple opportunistic infections in HIV-negative patients is exceedingly rare. We report a 29-year-old previously healthy female who presented with progressive cough, fever, and hemoptysis, culminating in life-threatening massive hemoptysis requiring intensive care. Imaging demonstrated diffuse tree-in-bud nodularity with necrotizing left upper lobe consolidation. Microbiologic evaluation revealed concurrent pulmonary tuberculosis, necrotizing methicillin-resistant *Staphylococcus aureus* pneumonia, and *Pneumocystis jirovecii* pneumonia. HIV testing was repeatedly negative. Subsequent immunologic evaluation demonstrated persistent isolated CD4 lymphopenia on serial testing, establishing a diagnosis of ICL. The patient was successfully treated with targeted antimicrobial therapy and supportive management, with stabilization of hemoptysis and clinical improvement. This case highlights a rare presentation of massive hemoptysis caused by synergistic pulmonary infections in the setting of previously undiagnosed ICL. The presence of *Pneumocystis jirovecii* pneumonia in an HIV-negative patient should prompt immediate evaluation for underlying cellular immune dysfunction. Early recognition of overlapping infectious etiologies and structured immune assessment are critical to timely diagnosis and optimal management in similar high-risk presentations.

## Introduction

Idiopathic CD4 lymphocytopenia (ICL) is a rare immunodeficiency characterized by persistent CD4⁺ T-cell depletion in the absence of human immunodeficiency virus (HIV) infection or other identifiable causes of immunosuppression [1]. It is formally defined as a CD4 T-cell count below 300 cells/mm³, or less than 20% of total T cells, on at least two measurements separated by six weeks in HIV-negative individuals without alternative causes of immune deficiency [1]. It is typically recognized only after opportunistic infections occur, and its pathogenesis remains unclear in most cases [1]. Pulmonary manifestations are common and include cryptococcosis, nontuberculous mycobacterial infections, and histoplasmosis [1]. Management focuses on treatment and prevention of opportunistic infections, as no definitive curative therapy exists [1,2].

Massive hemoptysis is most commonly associated with structural pulmonary diseases such as tuberculosis (TB), bacterial pneumonia, or malignancy, and becomes life-threatening when it causes airway compromise, respiratory failure, or hemodynamic instability [3-5]. In clinical practice, hemoptysis is often attributed to a single dominant pathology; however, overlapping infections may obscure diagnosis and delay targeted therapy [4-8].

*Pneumocystis jirovecii* pneumonia (PCP) in HIV-negative individuals is particularly concerning because it frequently signals underlying cellular immune dysfunction and is associated with more rapid clinical deterioration and higher mortality than HIV-associated disease [9-14].

We report a case of life-threatening massive hemoptysis caused by concurrent pulmonary TB, necrotizing methicillin-resistant *Staphylococcus aureus *(MRSA) pneumonia, and PCP infection, ultimately revealing ICL in a previously healthy young female. This rare presentation highlights the importance of evaluating cellular immune dysfunction in HIV-negative patients with multiple simultaneous pulmonary infections.

## Case presentation

We present a 29-year-old female from the Philippines with no significant past medical history and no chronic medication use who presented with one week of progressive cough, fever, and hemoptysis. She had worked as a housekeeping employee aboard a cruise ship for two years; her most recent voyage, from the Philippines to the Caribbean, had ended approximately four months before symptom onset.

Seven days before admission, she developed an intermittent productive cough with yellow sputum, which progressed within 48 hours to blood-streaked sputum and then frank hemoptysis (initially ~3 mL/24 hours), with fever to 38.2°C (100.8°F). She was treated empirically by the ship physician with azithromycin 250 mg (500 mg on day one, then 250 mg daily for four days), but symptoms worsened, and hemoptysis increased to ~40 mL/24 hours, prompting emergency department (ED) evaluation. On admission, she denied weight loss, night sweats, chills, fatigue, or malaise in the preceding months and reported no known sick contacts. She did not smoke cigarettes or use vaping products.

**Figure 1:**
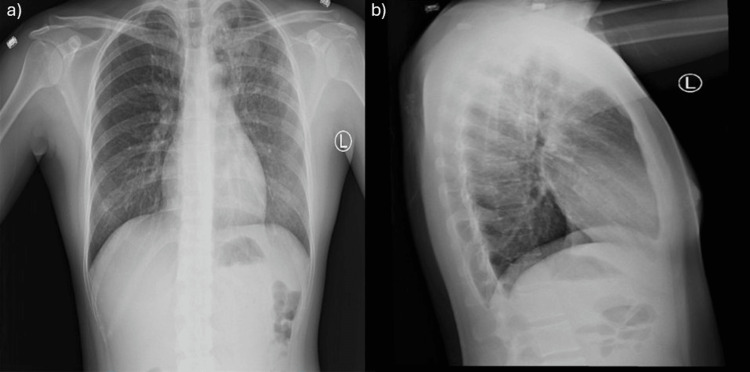
Chest X-ray. (a) Posteroanterior view demonstrating hazy opacification of the left upper lobe with air bronchograms and multifocal reticulonodular opacities, consistent with an infectious or inflammatory pulmonary process. (b) Lateral view confirming left upper lobe consolidation consistent with an infectious or inflammatory process such as pulmonary tuberculosis.

**Figure 2:**
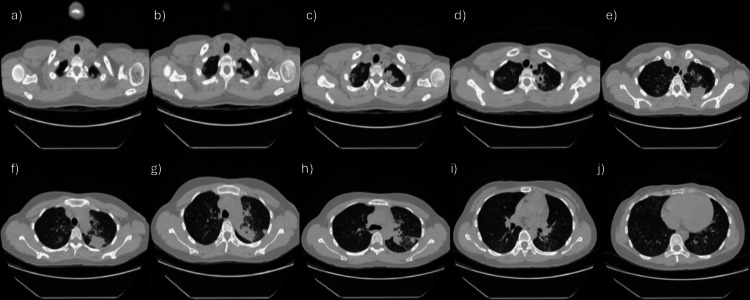
Chest CT without contrast. (a-c) Upper thoracic slices demonstrating cavitary lesions and nodular infiltrates predominantly involving the upper lobes. (d-f) Mid-lung slices demonstrating bilateral tree-in-bud and centrilobular nodularity consistent with endobronchial spread of infection. (g-j) Inferior thoracic slices demonstrating multifocal consolidation and diffuse nodular opacities with right apical pleural thickening.

Initial laboratory evaluation revealed a negative urine pregnancy test and an unremarkable urinalysis. Blood tests showed no leukocytosis but demonstrated elevated inflammatory markers and mild transaminitis (Tables 1-3). Chest radiography demonstrated a hazy left upper lobe opacity with air bronchograms and multifocal reticulation/nodularity (Figure 1). Computed tomography (CT) of the chest showed diffuse bilateral tree-in-bud and centrilobular nodularity with multifocal left upper lobe consolidations (largest 3.6 x 2.1 cm) with air bronchograms and right apical pleural thickening (Figure 2). These findings favored infectious pneumonia (bacterial vs. atypical), with pulmonary TB remaining a key diagnostic consideration. Based on the patient’s clinical presentation and imaging findings, the initial differential diagnosis included bacterial pneumonia, atypical pneumonia, pulmonary tuberculosis, and, less likely, a neoplastic process. The presence of tree-in-bud nodularity with upper-lobe predominance raised particular concern for tuberculosis or endobronchial spread of infection, prompting further microbiologic evaluation.

**Table 1:** Hematologic profile.

Date	White blood cells	Hemoglobin	Hematocrit	Platelets	Others
Day 1	7.82	13.4	39.4	335	Neutrophils 73%
Day 2 (morning)	8.01	12.7	36.6	331	Neutrophils 72.8%
Day 2 (afternoon)	—	11.7	34.6	—	—
Day 3	—	10.9	32	—	Down–trending hemoglobin
Reference range	4.0-11.0 ×10³/µL	12-17.5 g/dL	36-52 %	150-450 ×10³/µL	

**Table 2:** Metabolic profile.

Date	Sodium (Na^+^)	Potassium (K^+^)	Chloride (Cl^-^)	Carbon dioxide (CO₂)	Blood urea nitrogen (BUN)	Creatinine	Glucose	Calcium (Ca^2+^)	Aspartate aminotransferase (AST)	Alanine aminotransferase (ALT)	Alkaline phosphatase (ALP)	Albumin
Day 1	138	4.2	103	24	9	0.45	88	9.6	52	52	125	5
Day 2 (morning)	137	4.2	106	23	9	0.46	85	9.4	37	44	107	3.9
Day 2 (afternoon)	137	4.1	103	25	13	0.47	117	8.8	62	53	134	4.2
Reference range	135-145 mEq/L	3.5-5 mEq/L	98-106 mEq/L	22-29 mEq/L	7-20 mg/dL	0.6-1.3 mg/dL	70-99 mg/dL (Fasting)	8.5-10.5 mg/dL	10-40 U/L	7-56 U/L	44-147 U/L	3.5-5.0 g/dL

**Table 3:** Coagulation profile.

Date	Prothrombin time (PT)	International normalized ratio (INR)	Partial thromboplastin time (aPTT)
Day 1	10.6	1	33
Day 2	11.4	1	24
Reference range	11-13.5 seconds	0.8-1.1	25-35 seconds

Given the patient’s origin from a TB-endemic region and compatible imaging findings, airborne isolation precautions were implemented, and an infectious diagnostic workup was initiated. Empiric broad-spectrum antimicrobial therapy with cefepime and vancomycin was started. The patient became afebrile within 12 hours of antibiotic initiation, and the hemoptysis progressively decreased and ultimately resolved while she remained hemodynamically stable. Imaging findings were considered most consistent with atypical pneumonia with superimposed infectious bronchiolitis versus pulmonary TB, with a neoplastic process deemed less likely. The treatment was continued while evaluation for TB and atypical pathogens remained in progress.

Microbiologic evaluation was notable for the detection of *Staphylococcus aureus* on respiratory polymerase chain reaction (PCR) and an elevated *Mycoplasma pneumoniae* IgM titer (785). Interferon-gamma release assay (Quantiferon-TB Gold) returned positive, while HIV testing was negative. Sputum culture subsequently grew methicillin-resistant *Staphylococcus aureus* (MRSA), prompting discontinuation of cefepime and continuation of targeted therapy with vancomycin (Tables 4, 5). Oral azithromycin was added for atypical coverage given serologic evidence suggesting possible coinfection. Blood cultures were negative, and acid-fast bacilli smear and culture results were pending.

**Table 4:** Respiratory pathogen multiplex PCR panel.

Pathogen	Result	Pathogen	Result	Pathogen	Result
Staphylococcus aureus	Detected	Proteus	Not detected	Adenovirus	Not detected
Acinetobacter	Not detected	Pseudomonas aeruginosa	Not detected	Coronavirus (non–COVID)	Not detected
Enterobacter	Not detected	Serratia marcescens	Not detected	COVID-19	Negative
Escherichia coli	Not detected	Streptococcus agalactiae	Not detected	Human metapneumovirus	Not detected
Haemophilus influenzae	Not detected	Streptococcus pneumoniae	Not detected	Human rhinovirus/enterovirus	Not detected
Klebsiella aerogenes	Not detected	Streptococcus pyogenes	Not detected	Influenza A	Not detected
Klebsiella oxytoca	Not detected	Chlamydophila pneumoniae	Not detected	Influenza B	Not detected
Klebsiella pneumoniae	Not detected	Legionella pneumophila	Not detected	Parainfluenza	Not detected
Moraxella catarrhalis	Not detected	Mycoplasma pneumoniae	Not detected	Respiratory syncytial virus	Not detected

**Table 5:** Tuberculosis and atypical pathogen serologic testing.

Test	Value	Reference range	Interpretation
Quantiferon-TB Gold	-	Negative	Positive
TB1 antigen value	7.08	<0.35 IU/mL	Positive
TB2 antigen value	7.18	<0.35 IU/mL	Positive
Antigen - Nil	0.22	≤8.0 IU/mL	Valid test
Mitogen control	5.51	≥0.5 IU/mL	Adequate immune response
Mycoplasma IgM	785	<769 U/mL	Positive

Inflammatory markers demonstrated an erythrocyte sedimentation rate of 47 mm/hr, with mildly elevated lactate dehydrogenase (242 U/L) and normal serum lactate (1.2 mmol/L) (Table 6).

**Table 6:** Inflammatory panel.

Test	Value	Normal values
Erythrocyte sedimentation rate (ESR)	47	0-20 mm/hr
C-reactive protein (CRP)	0.9	<1.0 mg/dL
Lactate	1.2	0.5-2.0 mmol/L
Lactate dehydrogenase (LDH)	242	140-280 U/L

Bronchoscopy with bronchoalveolar lavage (BAL) was indicated for persistent hemoptysis with cavitary and tree-in-bud pulmonary lesions concerning for TB, necrotizing infection, or atypical pathogens, as noninvasive studies had not established a definitive diagnosis, and microbiologic confirmation was required to guide targeted therapy and infection control measures.

On hospital day four, while awaiting bronchoscopy, the patient developed massive hemoptysis consisting of a mix of fresh and partially clotted blood (~350 mL) with hypotension responsive to 1 L lactated Ringer’s and was transferred to the intensive care unit (ICU) due to respiratory distress. Endotracheal intubation and mechanical ventilation were recommended in case of recurrent bleeding or worsening respiratory compromise; however, the patient declined invasive ventilation. She was supported on a Venturi mask with a fraction of inspired oxygen (FiO₂) of 35% with oxygen saturation of ~97%. Arterial blood gas demonstrated pH of 7.46, partial pressure of carbon dioxide (pCO₂) of 33 mmHg, partial pressure of oxygen (pO₂) of 94 mmHg, and bicarbonate (HCO₃⁻) of 23 mmol/L, consistent with mild respiratory alkalosis (Table 7). Supportive therapy was initiated, including inhaled and intravenous tranexamic acid (1 g), intravenous corticosteroids, bronchodilators, antitussives, and close monitoring.

**Table 7:** Arterial blood gas. pCO₂: partial pressure of carbon dioxide; pO₂: partial pressure of oxygen; HCO₃-: bicarbonate; FiO₂: fraction of inspired oxygen.

Date	pH	pCO₂	pO₂	HCO₃^-^	O₂ Saturation	FiO₂	Device
Day 2	7.46	33	94	23	97	35	Venturi FiO₂ 35%
Reference range	7.35-7.45	35-45 mmHg	80-100 mmHg	22-26 mEq/L	95-100%	21% on room air	

**Figure 3:**
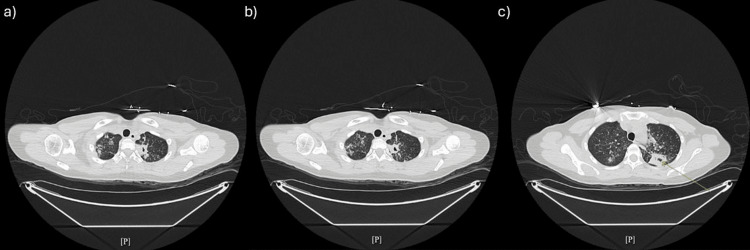
Dual-phase CT angiogram of the chest. (a) Axial CT slice demonstrating bilateral tree-in-bud micronodularity and cavitary changes. (b) Axial slice demonstrating necrotizing consolidation in the left upper lobe adjacent to segmental pulmonary arterial branches. (c) Contrast-enhanced image demonstrating the peribronchial consolidation with internal air foci and peripheral enhancement consistent with the likely bleeding source.

Dual-phase CT angiography was obtained to localize the bleeding source. Imaging redemonstrated diffuse bilateral tree-in-bud and centrilobular nodularity with peribronchial consolidation in the apicoposterior segment of the left upper lobe. The largest consolidation now showed internal air foci suggestive of necrosis and peripheral enhancement, located in close proximity to segmental pulmonary arterial vessels, likely representing the source of hemoptysis (Figure 3).

On hospital day six, after clinical stabilization of the patient’s hemoptysis and respiratory status, a diagnostic bronchoscopy BAL was performed. The procedure revealed severe diffuse airway erythema and friable mucosa without an identifiable active bleeding source or endobronchial lesion. BAL specimens were sent for acid-fast bacilli (AFB) smear, mycobacterial culture, cytology, and opportunistic infection testing (Table 8).

**Table 8:** Bronchoscopy and bronchoalveolar lavage.

Parameter	Result	Units/Notes
Fluid color	Pink	—
Appearance	Clear	—
WBC count	261	cells
Neutrophils	64	%
Lymphocytes	16	%
Eosinophils	0	%
Basophils	9	%
Mesothelial cells	0	%
Monocytes/macrophages	11	%

The AFB smear returned positive for mycobacteria, and additional testing identified PCP. A nucleic acid amplification test (NAAT/PCR, Xpert MTB/RIF (Mycobacterium tuberculosis/rifampicin)) performed on the respiratory sample detected *Mycobacterium tuberculosis* complex DNA with no evidence of rifampin resistance (no rpoB mutation detected), establishing the diagnosis of pulmonary TB (Table 9). The patient was promptly started on standard anti-TB therapy with the RIPE regimen (isoniazid with pyridoxine, rifampin, pyrazinamide, and ethambutol), planned for a two-month intensive phase followed by a four-month continuation phase with isoniazid and rifampin (total six months of therapy), along with vitamin B6. For PCP, trimethoprim-sulfamethoxazole (Bactrim) was initiated for a 21-day treatment course, with consideration for secondary prophylaxis depending on immune recovery. Intravenous vancomycin was discontinued, and the patient was transitioned to oral linezolid 600 mg every 12 hours to complete a total 14-day course for MRSA pneumonia. Bronchial artery embolization was deferred due to clinical improvement.

**Table 9:** Molecular testing results confirming pulmonary tuberculosis. NAAT: nucleic acid amplification test; MTB/RIF: Mycobacterium tuberculosis/rifampicin.

Test	Results
NAAT (Xpert MTB/RIF)	Positive for Mycobacterium tuberculosis complex
Rifampin resistance (rpoB mutation)	Not detected

An extensive immunodeficiency evaluation was initiated, given the coexistence of pulmonary TB and PCP in an HIV-negative adult. Immunoglobulin testing showed preserved humoral immunity (normal IgG and IgM) with elevated IgA and IgE consistent with reactive inflammation rather than antibody deficiency, and complement levels were normal. Additional studies, including lymphocyte subset analysis, BTK testing for X-linked agammaglobulinemia, adenosine deaminase (ADA) deficiency testing, chronic granulomatous disease oxidative burst assay, and a severe combined immunodeficiency (SCID) panel, were sent to assess for cellular and phagocytic defects (Table 10). In the absence of clear evidence of a congenital immunodeficiency and with a positive interferon-gamma release assay suggesting intact Th1 function, the findings favored secondary or transient immune dysfunction related to severe active TB. The patient was discharged in stable condition with these immunologic studies pending and arranged for close outpatient follow-up for completion of the evaluation, including repeat HIV testing in two to four weeks and assessment of CD4 lymphocyte count and immune recovery.

**Table 10:** Final immunology workup upon discharge. ADA: adenosine deaminase; CVID: common variable immunodeficiency.

Test/Study	Result	Purpose
Immunoglobulins (IgG, IgA, IgM, IgE)	IgG normal, IgM normal, IgA elevated, IgE elevated	Screens for humoral immunodeficiency (e.g., CVID, hyper-IgE syndromes)
Complement C3/Total complement	Normal (>60)	Evaluates complement pathway defects (encapsulated bacteria susceptibility)
X-linked agammaglobulinemia testing	Ordered	Detects BTK mutation → absent B-cells
Lymphocyte helper/suppressor panel (CD4/CD8 subsets)	Ordered	Assesses cellular immunity and T-cell deficiency
ADA deficiency testing	Ordered	Evaluates purine metabolism defect causing SCID
Chronic granulomatous disease panel	Ordered	Tests the neutrophil oxidative burst (NADPH oxidase function)
Severe combined immunodeficiency (SCID) panel	Ordered	Broad screening for combined T- and B-cell defects

Outpatient immunologic testing at four-week follow-up demonstrated persistent CD4 T-cell lymphopenia, with an absolute CD4 count of 182 cells/µL (18% of total lymphocytes) on initial measurement and 164 cells/µL (17%) on repeat testing performed six weeks later. CD8 counts were within normal range, yielding a reduced CD4/CD8 ratio, and total lymphocyte count was otherwise preserved. Repeat HIV antigen/antibody and HIV RNA testing remained negative, and evaluation for secondary causes of immunosuppression (including medication exposure, hematologic malignancy, autoimmune disease, malnutrition, and chronic systemic illness) was unrevealing. Immunoglobulin levels and complement function remained normal, and interferon-gamma release assay positivity confirmed intact Th1 cytokine production (Table 11).

**Table 11:** Four-week follow-up immunologic evaluation. ADA: adenosine deaminase; DHR: dihydrorhodamine; CGD: chronic granulomatous disease.

Test	Result	Interpretation
X-linked agammaglobulinemia (BTK mutation analysis)	Negative	Normal B-cell development; agammaglobulinemia excluded
Lymphocyte helper/suppressor panel (CD4/CD8 subsets)	CD4: 182 → 164 cells/µL (↓) ; CD8: normal ; CD4/CD8 ratio decreased	Persistent isolated CD4 lymphocytopenia
ADA deficiency testing	Normal ADA enzyme activity	SCID due to ADA deficiency was excluded
Chronic granulomatous disease oxidative burst assay (DHR test)	Normal neutrophil oxidative burst	NADPH oxidase function intact; CGD excluded
Severe combined immunodeficiency (SCID) panel	Negative for pathogenic variants	No combined T- and B-cell genetic immunodeficiency detected

The persistence of isolated CD4 lymphopenia on serial measurements in the absence of HIV infection or alternative etiologies established the diagnosis of idiopathic CD4 lymphocytopenia, explaining the patient’s susceptibility to concurrent pulmonary TB and PCP pneumonia. The patient continued regular follow-up with an infectious disease specialist in her home country for ongoing monitoring of CD4 counts and implementation of infection prophylaxis and long-term management of idiopathic CD4 lymphocytopenia.

## Discussion

This case illustrates life-threatening massive hemoptysis caused by synergistic pulmonary infection in the setting of previously undiagnosed ICL. The coexistence of pulmonary TB, necrotizing MRSA pneumonia, and PCP in an HIV-negative adult represents an exceptionally rare and clinically striking presentation and underscores the importance of structured immune evaluation when opportunistic infections occur outside classic risk groups.

The definition of massive hemoptysis has evolved from strict volume-based thresholds (300-600 mL per 24 hours) toward a functional definition based on physiologic impact, including airway obstruction, respiratory failure, or hemodynamic instability [4,15]. Mortality is most often related to airway compromise rather than blood loss alone, as even relatively small volumes of blood can rapidly impair ventilation and oxygenation through ventilation-perfusion mismatch [16]. In this case, the abrupt episode of approximately 350 mL of hemoptysis with associated hypotension met functional criteria for massive hemoptysis and required intensive care monitoring.

Computed tomography (CT) and bronchoscopy provide complementary diagnostic roles in the evaluation of hemoptysis. CT frequently identifies both the bleeding source and the underlying etiology and allows visualization of vascular abnormalities, whereas bronchoscopy is particularly useful for airway protection, localization of endobronchial lesions, and acquisition of microbiologic samples [15]. In this patient, CT angiography localized a necrotizing left upper-lobe consolidation adjacent to segmental pulmonary vessels, while bronchoscopy ultimately established the infectious etiologies through bronchoalveolar lavage.

Adjunctive therapies may be used to stabilize patients with acute hemoptysis. Tranexamic acid (TXA), particularly when administered via nebulization, has been reported to reduce bleeding duration, although evidence remains limited and its role is primarily supportive while definitive management is pursued [3,17]. In this case, inhaled and intravenous TXA were administered as temporizing measures during acute bleeding.

The tree-in-bud pattern observed on CT reflects endobronchial spread of infection with inflammation and plugging of small airways [18]. Although frequently associated with TB, this pattern is nonspecific and may occur with bacterial infection or aspiration [18-20]. In this patient, diffuse tree-in-bud nodularity with upper-lobe predominance and necrotizing consolidation created significant diagnostic complexity and ultimately reflected the presence of multiple concurrent infectious processes rather than a single pathogen.

Bacterial coinfection in pulmonary TB is well documented and has been associated with increased short-term mortality [21,22]. Chronic TB infection may also contribute to immune dysregulation through mechanisms including T-cell exhaustion and impaired cytokine responses [23-25]. These mechanisms may increase susceptibility to secondary bacterial infection, as likely occurred with the development of necrotizing MRSA pneumonia in this patient.

Hemoptysis in TB most commonly results from cavitary lung destruction and erosion of bronchial arteries. In this case, the abrupt onset of massive bleeding in the setting of necrotizing consolidation suggests a synergistic mechanism in which chronic cavitary damage from TB created structural vulnerability while MRSA-associated parenchymal destruction intensified vascular injury [15,24].

Diagnostic interpretation was further complicated by early laboratory findings, including a positive *Mycoplasma *IgM. Serologic testing for *Mycoplasma pneumoniae* has well-recognized limitations, including variable specificity and persistence of IgM following prior infection [26,27]. This illustrates the potential for early diagnostic anchoring when less reliable tests are interpreted before definitive microbiologic data become available.

The detection of PCP in an HIV-negative patient significantly broadened the differential diagnosis and raised concern for underlying cellular immune dysfunction. PCP in HIV-negative individuals typically progresses more rapidly and are associated with higher mortality compared with HIV-associated disease [9,28,29]. The identification of PCP therefore prompted further immunologic evaluation, which revealed persistent CD4 lymphopenia below 200 cells/µL.

In this case, the identification of PCP in an HIV-negative patient immediately broadened the differential toward underlying cellular immune dysfunction, given the absence of typical risk factors such as malignancy, autoimmune disease, transplant status, or immunosuppressive therapy [9,30]. The patient’s CD4 count below 200 cells/µL provided an explanation for the development of PCP, as the risk of opportunistic infections such as PCP increases substantially at this threshold [29]. Radiographically, PCP may produce ground glass opacities that overlap with TB and bacterial pneumonia, further complicating interpretation in the setting of triple infection [29]. Thus, PCP functioned not only as a diagnosis but as a clinical signal prompting deeper immune evaluation.

ICL is defined by persistent CD4 counts below 300 cells/µL on at least two measurements separated by six weeks in the absence of HIV infection or other causes of immunosuppression [1,2]. Although the pathogenesis remains incompletely understood, proposed mechanisms include impaired T-cell homeostasis, altered cytokine signaling, increased apoptosis, and rare genetic variants [1,14,31-34]. Unlike untreated HIV infection, CD4 counts in ICL often remain relatively stable over time rather than progressively declining. Management strategies are individualized and generally extrapolated from HIV guidelines, with prophylaxis and monitoring guided by CD4 level and clinical context [1,2].

TB epidemiology remains relevant in interpreting this case. In the United States, the majority of TB cases now occur in non-US-born individuals, with rates substantially higher than among US-born persons [35-38]. Many cases reflect reactivation of latent TB infection acquired in countries of origin, where prevalence varies widely [37,38]. Current recommendations support screening individuals from high-incidence regions regardless of time since arrival [37]. Foreign-born patients may also have higher rates of drug-resistant TB and may encounter barriers to timely diagnosis and treatment [36]. Given this patient’s origin from a TB-endemic region and occupational exposure on a cruise ship, early inclusion of TB in the differential diagnosis was epidemiologically appropriate and clinically justified.

## Conclusions

This case illustrates how life-threatening massive hemoptysis can arise from synergistic pulmonary infections in the setting of unrecognized cellular immune dysfunction. The concurrent presence of pulmonary tuberculosis, necrotizing MRSA pneumonia, and PCP in an HIV-negative patient is exceptionally rare and should prompt early consideration of idiopathic CD4 lymphocytopenia after exclusion of secondary causes. Overlapping radiographic features and initial attribution to a single pathogen may delay diagnosis and targeted therapy, increasing morbidity and mortality. Recognition of PCP in HIV-negative adults is particularly important, as it serves as a key clinical signal of underlying T-cell dysfunction. This case underscores the need for a multidisciplinary approach, early immune evaluation, and heightened diagnostic vigilance when multiple opportunistic pulmonary infections coexist outside classic risk groups.
